# Sociological analysis of the training of neonatal nurses in Minas Gerais (2000-2001)

**DOI:** 10.1590/0034-7167-2024-0508

**Published:** 2025-12-08

**Authors:** Isabelle de Souza Januaria, Cynthia Márcia Romano Faria Walty, Lélia Maria Madeira, Sérgio Deodato, Maria Itayra Padilha, Fernanda Batista Oliveira Santos

**Affiliations:** I Universidade Federal de Minas Gerais. Belo Horizonte, Minas Gerais, Brazil; II Fundação de Assistência Integral à Saúde, Hospital Sofia Feldman. Belo Horizonte, Minas Gerais. Brazil; III Universidade Católica Portuguesa. Lisboa, Portugal; IV Universidade Federal de Santa Catarina. Florianópolis, Santa Catarina, Brazil

**Keywords:** History of Nursing, Nurses, Neonatal, Education, Nursing, Graduate, Schools, Nursing, Sociology, Historia de la Enfermería, Enfermeras Neonatales, Educação de Postgrado en Enfermería, Facultades de Enfermería, Sociología

## Abstract

**Objectives:**

to analyze the creation and implementation of the first class of the Hospital Nursing Specialization Course focusing on Neonatal Nursing, between the years 2000 and 2001.

**Methods:**

a historical-social, descriptive study using documentary research, adopting established criteria for reporting qualitative research. It utilized eight documentary sources from the Memory Center of the Nursing School of the *Universidade Federal de Minas Gerais* (UFMG, Federal University of Minas Gerais) and the Sofia Feldman Hospital. The findings were analyzed under the framework of Eliot Freidson's sociology of professions.

**Results:**

two analytical categories emerged: "Initial efforts to enhance the training of specialist nurses in neonatology" and "Bringing ideas to life : implementation of the Hospital Nursing Specialization Course" : Details the concrete steps for turning the course into reality, from planning to execution.

**Conclusions:**

the first class of specialist neonatal nurses contributed to the development of nursing's own body of knowledge, enabling the acquisition of specialized expertise and the credentials necessary to support the profession's market reserve.

## RESUMEN

**Objetivos:**

analizar la creação e implementação de la primera clase del Curso de Especialização en Enfermería Hospitalaria con enfoque en Enfermería Neonatal, entre los años 2000 y 2001.

**Métodos:**

estudio histórico-social, descriptivo, utilizando investigação documental, adoptando criterios consolidados para relatar investigaciones cualitativas. Se emplearon ocho fuentes documentales del Centro de Memoria de la Escuela de Enfermería de la *Universidade Federal de Minas Gerais* (UFMG, Universidade Federal de Minas Gerais) y del Hospital Sofia Feldman. Los hallazgos se analizaron dentro del marco de la sociología de las profesiones de Eliot Freidson.

**Resultados:**

surgieron dos categorías analíticas: "Primeros movimientos para mejorar la formação de enfermeras especialistas en neonatología" y "Dando vida a las ideas : implementação del curso de Especialização en Enfermería Hospitalaria".

**Conclusiones:**

la primera clase de enfermeras especializadas en neonatología contribuyó al desarrollo de un cuerpo de conocimiento propio de la enfermería, permitiendo la adquisiação de experiencia especializada y las credenciales necesarias para apoyar la reserva de mercado de la profesião.

## INTRODUCTION

The Specialization Course in Hospital Nursing (CEEH), with an emphasis on Neonatal Nursing, offered by the School of Nursing at the *Universidade Federal de Minas Gerais* (UFMG, Federal University of Minas Gerais), was created in 2000 and began its activities in 2001. It represented a significant advancement in the state of Minas Gerais in terms of nursing training and care for high-risk newborns. This field had been experiencing growing investment and development in the state since the early 1990s^(1)^.

In 1994, Belo Horizonte's Health Department, through the *"Projeto Vida"*, reorganized maternal and neonatal care in the municipality. This included rigorous monitoring of newborns at higher risk of mortality^(2,3)^.

As a result of the restructuring of public and private services and social oversight assisted by municipal management, the Perinatal Commission of Belo Horizonte was created in 1994. Supported by the *Universidade Federal de Minas Gerais* (UFMG), the Perinatal Commission carried out a systematic evaluation of the city's hospitals, which led to the closure of low-quality maternity wards, investigation into maternal, infant, and fetal deaths, and the implementation of comprehensive care management, including the regulation of hospital care that was still in its early stages in Brazil^(2,3)^. This enabled the capital of Minas Gerais to become a national model for investigating maternal and neonatal mortality^(4)^.

The reorganization of perinatal care in Belo Horizonte not only restructured newborn care services but also created new possibilities for assistance. Before the creation of the Perinatal Commission, newborn care units primarily focused on maintaining and restoring neonatal vitality, preventing infections, and reducing morbidity and mortality^(2)^. These units adhered to a biomedical health model, centered on medical professionals' decisions, rather than on the entire healthcare team^(5,6)^.

In Brazil, the 1980s were marked by historical events that shaped the educational paths in health training to this day. Among these events were the 8th National Health Conference in 1986 and the promulgation of the Federal Constitution in 1988. Furthermore, during the 1990s, the approval of the Organic Health Law, No. 8080/1990, established the creation of the *Sistema Unificado de Saúde* (SUS, Unified Health System). The SUS model profoundly influenced the country's health system with its universal, comprehensive, equitable, decentralized care approach, alongside active social participation^(6)^.

Within the Brazilian social context, and specifically in the capital of Minas Gerais, Belo Horizonte, there arose a demand for neonatal nursing aligned with the real needs of high-risk pregnant women and newborns^(4)^. This brought prominence to two institutions: the School of Nursing at UFMG, a key player in the professionalization of nursing in the state^(7-9)^, and the Sofia Feldman Hospital (HSF)^(10)^.

In the 2000s, HSF and the School of Nursing at UFMG (EEUFMG) established a partnership to offer a specialization course aimed at training neonatal nurses in the city of Belo Horizonte. This course sought to provide nurses with the opportunity to deepen their specific knowledge and enhance their technical and administrative competence^(11)^.

In the field of historiography on specialized knowledge in nursing, obstetrics gained prominence in studies conducted in Minas Gerais^(9,12,13)^, viewed through the lens of Eliot Freidson's Sociology of Professions^(10,14-16)^. These studies^(10,12,14)^ observed that professional expertise (knowledge) was a key Freidsonian element in triggering professionalization in this area of specialization. Furthermore, while the field of neonatal nursing has received significant investment in the state, there are still few studies employing the historical-documentary method^(5,11)^.

It is believed that the demands of the neonatology field presented nursing with the challenge of developing its own specific knowledge, forming a pioneering movement that significantly shaped neonatology in the state and the country. This contributed to the initial steps toward professionalizing neonatal nurses, responding to both market needs and state regulation.

The assumption is that this specialization course in neonatology was offered due to a suppressed demand from society, representing a health requirement combined with the shortage of qualified professionals to meet local needs. Consequently, it contributed to the formation of specialized knowledge in neonatal nursing, leading health initiatives focused on the care of children in Minas Gerais.

The study is justified by providing valuable information regarding the process of training and recognizing neonatal nursing. This underscores the necessity of the profession's historiography for understanding the constitution of professional and, subsequently, institutional identity^(17)^.

## OBJECTIVES

To analyze the creation and implementation of the first class of the Specialization Course in Hospital Nursing with a focus on Neonatal Nursing, offered by the School of Nursing at UFMG in partnership with the Sofia Feldman Hospital, between the years 2000 and 2001. .

## METHODS

### Ethical Aspects

The study was conducted in accordance with national and international ethical guidelines. It utilized documentary sources available in the public collection of the Memory Center at the School of Nursing (CEMENF) of UFMG and was approved by the Research Ethics Committee of Sofia Feldman Hospital, whose approval document is attached to the present submission. The Free and Informed Consent does not apply since the research does not involve human subjects.

### Theoretical-methodological framework

The central idea of sociologist Eliot Freidson's proposal is that professions are defined by their ability to maintain or lose control over the terms, conditions, and content of their own work. Furthermore, Freidson characterizes a profession as one that possesses three key aspects: knowledge/expertise, self-regulation, and autonomy. Professions share a specialized body of knowledge and qualifications over which they hold jurisdiction. This jurisdiction is institutionalized through education in schools and universities, occupational control of practice within the labor market, and the requirement of credentials to perform licensed actions supported by the State^(15,16)^.

### Type of study

Study of the field of history, dimension of social history, domain of the history of nursing in interface with institutional history^(18)^. Approach: qualitative and documentary research. The study used the consolidated criteria for reporting qualitative research (COREQ) - version in Brazilian Portuguese^(19,20)^.

### Methodological procedures

To conduct this study, five steps were carried out: (1) formulation of the guiding research question, (2) definition of the objective, (3) document collection and data organization, (4) analysis of the collected data, and (5) interpretation and presentation of the results.

### Study setting

The study's setting was the city of Belo Horizonte, the capital of Minas Gerais, during the years 2000 and 2001. This was the location and period in which the first Neonatology Graduate Program, organized by the School of Nursing at UFMG in partnership with Sofia Feldman Hospital, was offered.

### Source of data

Documentary research was employed due to the availability of written sources that provided the foundation for the initial process of training neonatal nurses in Minas Gerais. Data regarding the training of neonatal nurses during the 2000:2001 period was analyzed. The year 2000 marked the initial events leading to the professional training of neonatal nurses by the School of Nursing at UFMG (EEUFMG), and 2001 represented the creation and implementation of the first class. However, it was necessary to access documents from 1997 to 1999, which explained why HSF was chosen as the field for offering the Specialization Course in Hospital Nursing (CEEH). The criterion for document selection was the mention or free discourse about the first CEEH offered by the EEUFMG.

### Data collection and organization

The document collection took place between April and July 2023, using written documents from the archives of the Memory Center of the School of Nursing at UFMG (CEMENF) and Sofia Feldman Hospital (HSF). These documentary collections were chosen for their representation of the historical context of the study settings, the EEUFMG and HSF. A document review form was used, containing the following research questions: "How was the creation and implementation of the Specialization Course in Hospital Nursing offered by EEUFMG at HSF (2000-2001) carried out?", "What relationships were established between HSF and EEUFMG for this creation?", and "To what extent did this course contribute to the professionalization of nurses working in the field of neonatology?". After reading the sources and condensing the data, the findings were organized into virtual folders, categorized by archive and year. Subsequently, eight key documents were selected for analysis, as outlined in Chart 1.

**Chart 1:** List of documents selected for analysis.

Document Identification	Name of the Source / Year	Consulted Archive
**Doc. 1**	Strategic Planning of the Sofia Feldman Hospital - 1997/1998.	HSF
**Doc. 2**	"Crescer" journal - 1998.	CEMENF
**Doc. 3**	Informative Bulletin of the Sofia Feldman Hospital - 1998.	CEMENF
**Doc. 4**	Document from the State Health Department of Minas Gerais - 1999.	CEMENF
**Doc. 5**	Plaque of the City Hall of Belo Horizonte - 2000.	HSF
**Doc. 6**	Strategic Planning of the Sofia Feldman Hospital - 2000/2001.	HSF
**Doc. 7**	Notice of the Specialization Course in Hospital Nursing - 2001.	CEMENF
**Doc. 8**	Regulations of the Specialization Course in Hospital Nursing - 2001.	CEMENF

Doc : Document; HSF : Sofia Feldman Hospital; CEMENF : Memory Center of the School of Nursing at UFMG.

### Data analysis

After reading and condensing, the findings were organized using the theoretical framework of the Sociology of Professions by Eliot Freidson, as it proves effective for analyzing professionalization processes and the development of specialized knowledge.

Thus, two analytical categories were outlined: "Initial efforts to enhance the training of neonatal nursing specialists" and "Bringing ideas to life : implementation of the Specialization Course in Hospital Nursing".

## RESULTS

### Initial efforts to enhance the training of neonatal nursing specialists

The Specialization Course in Hospital Nursing with a concentration in Neonatology was one of the work initiatives offered by the project titled Specialization Course in Hospital Nursing (CEEH) of EEUFMG. This course was created as a consequence of a healthcare system restructuring following the health reform, with the specific goal of contributing to the hospital network in Minas Gerais by training nurses from these services for more effective practices in specific fields (Doc. 7).

The CEEH collaborated with institutions such as HSF, Felício Rocho Hospital, *Fundação Hospitalar do Estado de Minas Gerais* (FHEMIG) and *Hospital das Clínicas* at UFMG, forming partnerships between these institutions and EEUFMG. Concentration areas like Neonatology, Intensive Care, Nephrology, and Transplants were established (Doc. 7).

For this reason, HSF was selected as the partner institution for training specialist nurses in neonatology. This choice was influenced by the leadership of both institutions and the fact that agreements already existed for nursing undergraduate and postgraduate programs between them, particularly for maternal and child care practice fields (Doc. 1; Doc. 2; Doc. 3; Doc. 7).

Serving a population of approximately 400,000 people, most of whom are low-income, the HSF, located in the North District on the outskirts of Belo Horizonte, the capital of Minas Gerais, stands out among various maternity hospitals in Brazil for its mission to humanize services for women and children, with full collaboration from the community (Doc. 3).

Since its inception, the HSF has relied on historical agents capable of audaciously rethinking individual care. The administrative directors of Sofia Feldman during the 1980s and 1990s implemented groundbreaking work within Brazilian health policy. The foundation of this health model was a community-based concept built on three pillars: social control exercised by the Community Association of Friends and Users (ACAU), which brought humanization to care; voluntary work through projects like "substitute mothers" and "community doulas", showcasing the community's interest in actively participating; and oversight through the figure of the ombudsman, enabling society itself to monitor its hospital (Doc. 1; Doc. 3).

This healthcare model was groundbreaking and enabled HSF to become a site of significant investments in various aspects, starting with excellence in services focused on pregnant women and humanized childbirth. This led to improvements in the quality of care provided and allowed the hospital to achieve titles and awards, such as the Galba de Araújo Award, granted by the Ministry of Health (MS) for humanization in childbirth care (1990); the designation as the "First Baby-Friendly Hospital" in the state of Minas Gerais, recognized by the United Nations Children's Fund (1995); and the Citizenship Award, given by the Ministry of Health (1997) (Doc. 3).

HSF was also ranked as the second-best maternity hospital in Brazil (1998), evaluated based on factors such as the cesarean rate, humanization, prenatal care, hospital infection rates, an in-house medical team, and the emergency structure and support services (Doc. 2).

These achievements are linked to an institutional visionary condition regarding the appreciation of multiprofessional work by having care provided by a team composed of a nurse, doctor, social worker, psychologist, pharmacist, nutritionist, and other nursing professionals. Additionally, by having an on-call team consisting of a nurse, obstetricians, pediatrician, anesthesiologist, and community doula (Doc. 3).

The maternity ward prioritized the focus on risk and the appropriate use of technologies by offering care during childbirth from a holistic and interdisciplinary perspective, considering that there is no conception of illness, but rather of life. This approach challenged the technicist and biomedical model present in various hospitals in Minas Gerais (Doc. 3).

Highlighting the knowledge and responsibilities assigned to the nurses at HSF, the hospital aimed to offer more humanized care, rescuing the natural and physiological character of childbirth, allowing women to actively participate in the process. One of the strategies used to achieve this was to leave prenatal care and high-risk delivery assistance under the nurse's responsibility (Doc. 3).

The model developed and refined over the decades had a positive impact on care indicators. In this regard, HSF has been providing childbirth assistance carried out by nurses since 1982, accounting for approximately 80% of deliveries, and a rooming-in system in 100% of hospitalizations. Since 1988, it has been a reference center for family planning for the MS (Ministry of Health), and since 1989 it has been part of the *"Projeto Canguru"* (Kangaroo Project) in Brazil. During all hospital stays, women receive support from their partners and volunteer doulas during prenatal, childbirth, and postpartum periods (Doc. 3; Doc. 4).The maternity ward had an average of 400 births per month, of which 15% were high-risk patients (Doc. 3). It also faced challenges concerning the rising cesarean rate, which was nearing the limit recommended by the MS.

From the moment HSF began to establish itself as a reference in maternal care, the need for continuity of care for at-risk newborns also emerged. Until that time, HSF had pediatric beds, and since 1998, a project was underway to create a high-risk nursery, following the model of joint hospitalization (Doc. 1; Doc. 3). Until 1999, newborns requiring critical care were referred to the Hospital Odilon Behrens. Based on the indicators, this approach was costly and could cause greater harm to the referred mothers and children, going against the institution's proposed healthcare model and the emphasis on humanization (Doc. 1; Doc. 6).

Given the scale of HSF's development, it became necessary for the hospital to take responsibility for the care of all neonatal patients, as the partnership with the Hospital Odilon Behrens involved higher costs and generated additional expenses for referring all patients (Doc. 6).

Given the quality of healthcare provided by HSF to the population of Minas Gerais, the city hall of Belo Horizonte, through its partnership with HSF, delivered 10 neonatal intermediate care unit beds to the community. These beds were designated for newborns requiring specialized care, marking the establishment of the first space dedicated to specialized care for at-risk newborns (Doc. 5).

At that time, EEUFMG, an institution with nearly 70 years of history, maintained its leadership in key processes for the professionalization of nursing in Minas Gerais. Focused on developing and producing knowledge unique to the profession, and despite being influenced by the political, economic, and specific legislative contexts, EEUFMG sought to meet the state's demands regarding neonatal health (Doc. 7).

### Bringing ideas to life : implementation of the Specialization Course in Hospital Nursing

EEUFMG proposed the creation of the Specialization Course in Hospital Nursing (CEEH), based on the principle of learning through practice. Its objective was to contribute to the development of nursing professionals in the hospital field while simultaneously reflecting on their care practices for neonatal patients (Doc. 7).

The CEEH's methodology was grounded in the principles of problem-based pedagogy. The course was offered in a hybrid format, combining in-person and remote components to accommodate the nurses' experiences and daily work, following the principles of integrating teaching and work (Doc. 7).

The course employed various teaching techniques, such as seminars, conferences, lectures, small group activities, clinical and managerial case discussions, and independent studies, aiming to foster active student participation. Independent study activities included reading, analyzing bibliographic material, and engaging in activities and practices within each student's predefined work environment (Doc. 7).

The faculty was composed of twenty-three nurses, seven of whom held doctoral degrees, fifteen held master's degrees, and one had a specialist degree. All faculty members were involved in teaching the course"”eighteen focused solely on mentorship, while eighteen were engaged in both teaching and mentoring the students (Doc. 7).

The course's faculty were predominantly graduates of UFMG's master's program in nursing (Doc. 7), which strengthened their connection to the institution and their commitment to advancing knowledge in the field of nursing in Minas Gerais.

The CEEH had a total workload of 420 hours, equivalent to 28 credits, and was designed to be completed within a maximum of twelve months. Its administration was overseen by the EEUFMG Graduate Collegiate, with faculty drawn from three EEUFMG departments: Basic Nursing, Applied Nursing, and Maternal-Child and Public Health Nursing, highlighting the integration of diverse expertise among the instructors.

Nurses who chose the concentration area in neonatology had their curriculum organized into two components: a common core and a specialized core. The common core had a theoretical workload of 210 hours and covered a set of topics necessary for shaping the profile of a specialist nurse in pedagogical, clinical, and managerial fields. It consisted of five modules: Pedagogical Training, Evaluating the Client's Health Status, Research Methodology, Breaking the Chain of Disease Transmission, and Managing Nursing Care (Doc. 7).

These subjects collectively provided a body of knowledge aimed at promoting the development of teaching and learning activities in the course, while contributing to the systematic application of knowledge in professional nursing practice and the preparation of future specialists in neonatology (Doc. 7; Doc. 8).

The specialized core, titled "Neonatal Nursing", used HSF as the setting for practical care training. It encompassed theoretical knowledge in neonatology, delving deeper into care methodologies to support students in their chosen area. The specialized core also had a total workload of 210 hours (Doc. 7; Doc. 8).

In its syllabus, the course with an emphasis on Neonatal Nursing sought to address the biopsychosocial characteristics and needs of newborns and their families, as well as the demands for comprehensive nursing care (Doc. 7; Doc. 8). The course aimed to analyze the most common conditions affecting newborns, focusing on their pathophysiology and the intervention measures needed to apply nursing care to term, preterm, and post-term newborns. This included care in the delivery room, joint accommodations, the neonatal unit, as well as their outpatient follow-up (Doc. 7; Doc. 8).

From this perspective, when the three nursing departments of EEUFMG joined forces to offer a specialization course, they were striving to promote and invest in an interdisciplinary education for nurses. This initiative resulted in the graduation of ten neonatal nursing specialists and marked EEUFMG's pioneering role in providing postgraduate education in neonatal nursing in Minas Gerais.

## DISCUSSION

This study utilized the framework of the Sociology of Professions by Eliot Freidson^(15,16)^. The historical construction of a knowledge field for neonatal nursing involves various struggles undertaken by the profession. In this context, it is worth noting that professions are occupations that assume dominant positions in the division of labor, controlling and determining the essence of their own work, thereby being autonomous and self-regulated^(15,16,21)^.

Freidson argues that the expansion and consolidation of a profession occur through its professional organization and the professionalism of its members within this formal occupational activity. Professionalization, therefore, is characterized by three key factors: autonomy, expertise, and credentialism^(15,16,22)^.

Autonomy refers to a profession's control over the technical aspects of its work, establishing what is essential and unique to the profession. This factor becomes evident in environments where the division of labor among professions exists, with each area having its own specificities and responsibilities^(15,16,21,22)^.

The autonomy of nurses at Sofia Feldman Hospital (HSF) is an institutional commitment. Since its inception, the institution has paid close attention to the healthcare needs of the population, entrusting prenatal and high-risk childbirth care to nurses. Therefore, it is important to consider that achieving autonomy depends on factors such as the ability to make independent decisions, being free from coercion, having rational and reflective thinking, possessing adequate knowledge and information, and the right to control one's own work^(23,24)^.

Freidson's writings classify nursing as an occupation or even as a paraprofessional activity^(15,16)^, relying on the determinations of another professional to carry out its practice^(24)^. According to Freidson, traditionally, most nurses lack autonomy because they work in hospitals structured around the biomedical model, depending on medical orders and requirements, which increases the potential for conflict^(15,16)^. However, this perspective is not agreed upon in this case, as the decision-making involved in establishing the Specialization Course demonstrates an exercise of autonomy, credentialism, and expertise. The course organizers successfully obtained authorization and implemented the program with distinction.

When analyzing the landscape of the 1990s, HSF stood out as a pioneering institution that believed in and invested in the work of nurses. It became a space that facilitated and fostered forms of resistance capable of weakening traditional hegemony, allowing the nurses working there to redefine their governance and promote their autonomy. This approach ensured safe and high-quality care.

However, some studies highlight that professional autonomy for nurses in intensive care environments remains limited and, at times, suppressed within healthcare organizations, particularly in institutions where the biomedical model is strongly enforced. In such settings, nurses' autonomy is constrained and often dependent on medical professionals' decisions, the fragile establishment of a distinct body of knowledge within the nursing profession, and the increasing technical division of labor in healthcare and nursing^(25-28)^.

At HSF, nurses' autonomy was encouraged within the institution's collaborative spaces, beginning with the existence of a multidisciplinary and interdisciplinary team composed of nurses, physicians, social workers, psychologists, pharmacists, nutritionists, and other nursing professionals. Additionally, there was an on-call team that included nurses, obstetricians, pediatricians, anesthesiologists, and community doulas. This team composition allowed and contributed to enabling nurses, through credentialism ensured by the State and professional bodies, to become instruments of empowerment at Hospital Sofia Feldman.

It is worth emphasizing that HSF's ability to carry out these activities stemmed from its understanding that strategies for transforming and improving healthcare practices are built through dialogue among all actors involved in childbirth and neonatal care. The collaborative coexistence of diverse professions played a pivotal role in fostering these practices.

Expertise is the specialized knowledge unique to a profession, which forms the formal essence of learning for experts in professional schools. This expertise is shaped by specific training under formal regulations^(15,16,21,22)^. In this context, the creation of the Specialization Course in Hospital Nursing (CEEH) with a focus on neonatal nursing aimed to develop a specific body of knowledge for practice in a defined setting.

HSF exemplifies such a space - a public hospital recognized for its care for women and newborns across various modes of care^(29)^. Selecting this environment as the practical field for a specialization course training neonatal nurses aimed to equip them with the knowledge necessary to address the real health needs of the population and improve professional working conditions. Essentially, it sought to form a group of neonatology experts who could serve as catalysts for training other professionals. The CEEH prioritized preparing nurses to engage directly with the population, a goal reinforced by the institution's international title as a "Baby-Friendly Hospital" since 1995. This model of healthcare is rooted in community-based principles, with one of its three pillars focused on social accountability^(29)^. The course modules "Pedagogical Training" and "Evaluating the Client's Health Status" reflected this approach in 75 hours of theoretical classes.

Moreover, expertise is expressed by consulting professionals who, in some way, hold a monopoly over the exercise of their specialized knowledge and are tasked with solving practical issues presented by society, thereby attracting the spontaneous trust of those who consult them^(15,16,22)^. This knowledge has enabled the recognition of the nurse's status quo among the assisted population, making the professional autonomous and legitimized by the credentials granted.

Hence, expertise ensures the specificity of know-how and provides autonomy in defining problems under the professional's domain. Additionally, it establishes conditions for structuring and organizing the foundations for independent professional practice^(21,23,24)^.

In this regard, the expertise developed within knowledge-production environments offers neonatal patients protection through specialized care provided by nurses, as supported by the literature^(30,31)^. The development of scientific knowledge in neonatology as a nursing field has contributed to gaining spaces within the institution and the profession, which were crucial for achieving titles and honors tied to the quality of care produced by HSF's nurses.

The role of EEUFMG is highlighted as a key driver of professionalization processes both in Minas Gerais and across Brazil, pioneering efforts to promote the autonomy of nurses^(5)^. This was supported by the institution's development of specific knowledge to address socio-sanitary demands effectively. Equally notable is HSF's innovative actions in the healthcare sector, emphasizing its engagement with the community, which enabled the development of initiatives aimed at addressing the actual needs of the population.

### Study limitations

This study is set within the context of a Brazilian capital, featuring a maternity hospital and a nursing school that serve as state-wide references. The interpretation of sources from other regions might reveal health circumstances and healthcare professional training processes that differ from those experienced in major urban centers. Furthermore, the absence of research in additional archives, such as those of the Public Library, may have limited the scope of information regarding the training of neonatal nurses during the studied period.

### Contributions to the fields of healthcare, nursing, or public policies

This marks the first investment in the historiographic research of neonatal nursing in the state of Minas Gerais. The findings illuminate historical facts that support an understanding of the leading roles played by EEUFMG and HSF in the context of professionalizing nursing in Minas Gerais and Brazil. This research contributes to documenting and showcasing a pioneering model of nurse training dedicated to addressing the healthcare needs of the population.

By making public the configuration of a specialized field of nursing knowledge in Minas Gerais, this study fosters recognition of nurses as historical agents shaping the professionalization process. It nurtures a sense of belonging and a collective professional identity rooted in a shared knowledge base and the ongoing fight for the appropriation and protection of this space.

The historiography of establishing and implementing nursing services in specialized contexts, beyond preserving memory, also strengthens professional and institutional identities. The historical experience of a group or society serves as valuable references for the present. While health historiography encompasses a body of research^(29-31)^, this study's unprecedented focus on neonatal nursing in Minas Gerais and at the national level stands out as a significant contribution.

## FINAL CONSIDERATIONS

The first offering of a course for the training of neonatal nursing specialists by EEUFMG, in partnership with HSF, marked a milestone in the history of nursing in Minas Gerais and for both institutions. The course contributed to the advancement of the profession, the implementation of innovative practices, and the empowerment of nurses in building nursing-specific knowledge. It facilitated the acquisition of specialized expertise and the credentials necessary to support the professionalization and exclusivity of the nursing workforce in the job market.

## Data Availability

The research data are available only upon request.
