# Supplementary material for: Sociological analysis of the training of neonatal nurses in Minas Gerais (2000-2001)
Source: Rev Bras Enferm. 2025 Dec 8;78(4):e20240508. doi: 10.1590/0034-7167-2024-0508 (PMC12716809; doi:10.1590/0034-7167-2024-0508)
Supplement: Translation

# Translation: Análise sociológica da formação de enfermeiras neonatologistas em Minas Gerais (2000 – 2001)

**DOI:** 10.1590/0034-7167-2024-0508pt

**Authors:** Isabelle de Souza Januaria, Cynthia Márcia Romano Faria Walty, Lélia Maria Madeira, Sérgio Deodato, Maria Itayra Padilha, Fernanda Batista Oliveira Santos

**Affiliations:** I Universidade Federal de Minas Gerais, Belo Horizonte, Minas Gerais, Brasil; II Fundação de Assistência Integral à Saúde, Hospital Sofia Feldman. Belo Horizonte, Minas Gerais. Brasil; III Universidade Católica Portuguesa. Lisboa, Portugal; IV Universidade Federal de Santa Catarina. Florianópolis, Santa Catarina, Brasil

**Keywords:** História da Enfermagem, Enfermeiros Neonatologistas, Educação em Enfermagem de Pós-Graduação, Escolas de Enfermagem, Sociologia

## Abstract

**Objetivos:**

analisar a criação e implantação da primeira turma do Curso de Especialização em Enfermagem Hospitalar com foco em Enfermagem Neonatal, entre os anos de 2000 e 2001.

**Métodos:**

estudo histórico-social, descritivo, pesquisa documental, adotados os critérios consolidados para relatar a pesquisa qualitativa. Utilizou oito fontes documentais do Centro de Memória da Escola de Enfermagem da Universidade Federal de Minas Gerais e do Hospital Sofia Feldman. Os achados foram analisados sob o arcabouço da sociologia das profissões de Eliot Freidson.

**Resultados:**

duas categorias analíticas: "Primeiros movimentos para aprimoramento da formação de enfermeiras especialistas em neonatologia" e "Dando vida à s ideias : implantação do curso de Especialização em Enfermagem Hospitalar".

**Conclusões:**

a primeira turma de enfermeiras especialistas em neonatologia contribuiu com a construação do conhecimento próprio de enfermagem, possibilitando a obtenação do conhecimento especializado e das credenciais necessárias para amparar a reserva de mercado de trabalho.

## INTRODUÇÃO

O Curso de Especialização em Enfermagem Hospitalar (CEEH) com à ªnfase em Enfermagem Neonatológica, ofertado pela Escola de Enfermagem da Universidade Federal de Minas Gerais (EEUFMG), foi criado em 2000 e iniciou suas atividades em 2001, representando, no à ¢mbito do estado de Minas Gerais, um avanço na formação em enfermagem e na assistência aos recém-nascidos de alto risco. Este era um campo que estava em crescente apropriação e investimento no estado desde o início da década de 1990^(1)^.

Em 1994, a Secretaria de Saúde de Belo Horizonte, por meio do Projeto Vida, realizou uma reorganização da assistência à gestante e ao recém-nascido no município, o que incluía o acompanhamento rigoroso dos recém-nascidos com maior risco de óbito^(2,3)^.

Em decorrência desta reestruturação dos serviços públicos, privados, e no controle social, apoiada na gestão municipal, criou-se em 1994 a Comissão Perinatal da cidade de Belo Horizonte. Amparada pela Universidade Federal de Minas Gerais (UFMG), a Comissão Perinatal elaborou uma avaliação sistemática dos hospitais da capital, o que acarretou o fechamento das maternidades de baixa qualidade, a investigação dos óbitos maternos, fetais, infantis e uma gestão do cuidado integral, incluindo a regulação da assistência hospitalar ainda incipiente no país^(2,3)^. Isto permitiu que a capital mineira se tornasse modelo nacional em investigação à mortalidade materna e neonatal^(4)^.

A reorganização da assistência perinatal em Belo Horizonte permitiu uma reestruturação do cenário de atendimento ao recém-nascido e também novas possibilidades de atendimento. Até a criação da Comissão Perinatal, as unidades de atendimento ao recém-nascido tinham por finalidade a manutenação e restauração das condições de vitalidade do neonato, a prevenação de infecções, e a diminuiação da morbimortalidade^(2)^. Tinham aderência ao modelo biomédico de saúde, centralizado nas decisões do profissional médico, e não na equipe de saúde como um todo^(5,6)^.

No país, os anos 1980 são marcados por eventos históricos responsáveis pelos caminhos curriculares que a formação em saúde atende até os dias atuais. Dentre eles, a realização da 8Âª Conferência Nacional de Saúde, no ano de 1986, e a promulgação da Constituiação Federal, no ano de 1988. Não obstante esses eventos, na década de 1990 houve a aprovação da Lei Orgânica da Saúde, nÂ° 8080/1990, que explicitou a criação do Sistema Ãšnico de Saúde (SUS). O modelo de saúde do SUS impactou todo o processo de conduação da saúde no país do ponto de vista do cuidado universal, integralizado, equânime, descentralizado, e com participação social ativa^(6)^.

Diante da conjuntura social brasileira e, em especial, da capital de Minas Gerais, Belo Horizonte, criou-se uma demanda para a enfermagem neonatológica que deveria estar em consonância com as reais necessidades da gestante e do recém-nascido de alto risco^(4)^, ganhando assim destaque duas instituições: a Escola de Enfermagem da UFMG, protagonista no processo de profissionalização da enfermagem no estado^(7-9)^, e o Hospital Sofia Feldman (HSF)^(10)^.

Nos anos 2000, o HSF e a Escola de Enfermagem da UFMG instituíram uma parceria para o oferecimento de um curso de especialização para a formação de enfermeiras neonatologistas na cidade de Belo Horizonte. Este curso tinha como objetivo proporcionar à s enfermeiras condições para aprofundarem saberes específicos e aprimorarem sua competência técnico-administrativa^(11)^.

No à ¢mbito da historiografia do conhecimento especializado em enfermagem, a obstetrícia ganhou destaque em estudos em Minas Gerais^(9,12,13)^ sob o prisma da Sociologia das Profissões de Eliot Freidson^(10,14-16)^. Nestes estudos^(10,12,14)^, observou-se que a *expertise* (conhecimento) profissional foi elemento-chave freidsoniano para deflagrar a profissionalização nesta area de especialização. Outrossim, o campo da enfermagem neonatológica possui grandes investimentos no estado, mas há poucos estudos utilizando o método histórico-documental^(5,11)^.

Acredita-se que as exigências do campo da neonatologia trouxeram para a enfermagem o desafio de construir um conhecimento próprio e específico, formando-se um movimento vanguardista importante para a conformação da neonatologia no estado e no país, o que contribuiu para os primeiros passos da profissionalização de enfermeiras neonatologistas diante da necessidade do mercado e da regulação do Estado.

Partiu-se do pressuposto de que este curso de especialização em neonatologia foi ofertado a partir de uma demanda reprimida da sociedade, sendo uma requisiação sanitária somada à falta de profissionais qualificados para atender à s necessidades locais. Posto isto, contribuiu para a conformação de conhecimento especializado em enfermagem neonatológica, protagonizando ações em saúde no cuidado à criança mineira.

O estudo se justifica por trazer informações relevantes sobre o processo de formação e reconhecimento da enfermagem neonatal. Isto mostra o quanto a historiografia da profissão se faz necessária para o entendimento da constituiação da identidade profissional e, por conseguinte, institucional^(17)^.

## OBJETIVOS

Analisar a criação e implantação da primeira turma do Curso de Especialização em Enfermagem Hospitalar com foco em Enfermagem Neonatal, da Escola de Enfermagem da Universidade Federal de Minas Gerais em parceria com o Hospital Sofia Feldman, entre os anos de 2000 e 2001.

## MÃ‰TODOS

### Aspectos éticos

O estudo foi conduzido de acordo com as diretrizes de ética nacionais e internacionais, abordou fontes documentais disponíveis no acervo público do Centro de Memória da Escola de Enfermagem da Universidade Federal de Minas Gerais (CEMENF) e foi aprovado pelo Comitê de Ética em Pesquisa do Hospital Sofia Feldman (HSF), cujo parecer está anexado à presente submissão. O Consentimento Livre e Esclarecido não se aplica, pois, a pesquisa não envolve seres humanos.

### Referencial teórico-metodológico

A ideia central da proposta do sociólogo Freidson é que as profissões se baseiam no modo como elas são capazes ou perdem o controle sobre os termos, as condições e o conteúdo do seu próprio trabalho. Ademais, Eliot Freidson caracteriza uma profissão como aquela que detém três aspectos: o conhecimento/*expertise*, a autorregulação e a autonomia. As profissões compartilham um corpo especializado de conhecimentos e qualificações sobre o qual têm jurisdiação, e cuja credencial ocorre por meio da institucionalização em escolas e universidades, do controle ocupacional da prática no mercado de trabalho, e pela exigência de credenciais para executar ações licenciadas e respaldadas pelo Estado^(15,16)^.

### Tipo de estudo

Estudo do campo da história, dimensão da história social, domínio da história da enfermagem em interface com a história institucional^(18)^. Abordagem: pesquisa qualitativa e documental. Utilizou-se os critérios consolidados para relatar a pesquisa qualitativa (COREQ) - versão em português falado no Brasil^(19,20)^.

### Procedimentos metodológicos

Para a elaboração deste estudo, foram percorridas cinco etapas: (1) formulação da questão norteadora da pesquisa, (2) definiação do objetivo, (3) coleta documental e organização dos dados, (4) análise dos dados encontrados e (5) interpretação e apresentação dos resultados.

### Cenário do estudo

O cenário de estudo foi a cidade de Belo Horizonte, capital de Minas Gerais, nos anos 2000 e 2001, local e período em que ocorreu a organização e oferta do primeiro curso de Pós-Graduação em Neonatologia da Escola de Enfermagem da UFMG em parceria com o hospital Sofia Feldman.

### Fonte de dados

Foi utilizada a pesquisa documental pela disponibilidade de fontes escritas que fundamentaram o início do processo formativo de enfermeiras neonatologistas em MG. Foram analisados os dados referentes à formação de enfermeiras neonatais no período de 2000 e 2001. O ano de 2000 marca os primeiros acontecimentos para a formação profissional de enfermeiras neonatais pela EEUFMG, e o ano de 2001 é o ano da criação e implantação da primeira turma. Contudo, foi necessário acessar documentos de 1997 a 1999 que retrataram o porquê de o HSF ter sido o campo escolhido para a oferta do CEEH. O critério para seleação de documentos foi a menação ou discurso livre acerca do primeiro Curso de Especialização em Enfermagem Hospitalar ofertado pela EEUFMG.

### Coleta e organização dos dados

A coleta documental foi feita no período de abril a julho de 2023, a partir de documentos escritos encontrados nos acervos do CEMENF e do HSF. Essas coleções documentais foram escolhidas por serem representativas da história dos cenários deste estudo, a Escola de Enfermagem da UFMG e o HSF. Utilizou-se uma ficha documental contendo as seguintes perguntas de pesquisa: "Como se deu a criação e a implantação do curso de especialização em Enfermagem Hospitalar ofertado pela EEUFMG no HSF (2000-2001)?", "Quais foram as relações instituídas entre o HSF e a EEUFMG para esta criação?", e "Este curso apoiou em que medidas a profissionalização das enfermeiras atuantes na área de neonatologia?". Após a leitura das fontes e condensação dos dados, os achados foram organizados em pastas virtuais, separadas por acervo e por ano. Assim, foram selecionados oito documentos chave para análise, conforme Quadro 1.

**Quadro 1:** Relação dos documentos selecionados para análise

Identificação do documento	Nome da fonte/Ano	Acervo consultado
**Doc. 1**	Planejamento Estratégico do Hospital Sofia Feldman - 1997/1998.	HSF
**Doc. 2**	Revista Crescer - 1998.	CEMENF
**Doc. 3**	Boletim Informativo do Hospital Sofia Feldman - 1998.	CEMENF
**Doc. 4**	Ofício da Secretaria do Estado de Saúde de Minas Gerais - 1999.	CEMENF
**Doc. 5**	Placa prefeitura de Belo Horizonte - 2000.	HSF
**Doc. 6**	Planejamento Estratégico Hospital Sofia Feldman - 2000/2001.	HSF
**Doc. 7**	Edital Curso de Especialização de Enfermagem Hospitalar - 2001.	CEMENF
**Doc. 8**	Regulamento do Curso de Especialização em Enfermagem Hospitalar - 2001.	CEMENF

Doc : Documento; HSF : Hospital Sofia Feldman; CEMENF : Centro de Memória da Enfermagem.

### Análise dos dados

Após a leitura e condensação, os achados foram organizados considerando o referencial teórico da Sociologia das Profissões segundo Eliot Freidson, pois este se mostra assertivo para análises dos processos de profissionalização e construação de saberes especializados.

Assim, foram delineadas duas categorias analíticas: "Primeiros movimentos para aprimoramento da formação de enfermeiras especialistas em neonatologia" e "Dando vida à s ideias : implantação do curso de Especialização em Enfermagem Hospitalar".

## RESULTADOS

### Primeiros movimentos para aprimoramento da formação de enfermeiras especialistas em neonatologia

O Curso de Especialização em Enfermagem Hospitalar com área de concentração em Neonatologia foi uma das frentes de trabalho ofertadas pelo projeto denominado Curso de Especialização em Enfermagem Hospitalar (CEEH) da EEUFMG. Este curso foi criado como consequência de uma reestruturação do sistema de saúde após a reforma sanitária, com o propósito específico de contribuir com a rede hospitalar de Minas Gerais, capacitando enfermeiras destes serviços para uma prática mais resolutiva em áreas de atuação específicas (Doc. 7).

O CEEH articulou-se com instituições como o Hospital Sofia Feldman (HSF), Hospital Felício Rocho, Fundação Hospitalar do Estado de Minas Gerais e Hospital das Clínicas da UFMG, estabelecendo parcerias entre estas instituições e a EEUFMG. Foram estruturadas áreas de concentração como Neonatologia, Terapia Intensiva, Nefrologia e Transplantes (Doc. 7).

Por esse motivo, o HSF foi a instituiação parceira eleita para a formação de enfermeiras especialistas em neonatologia. Esta escolha tem relações com as lideranças de ambas as instituições envolvidas, e com o fato de já existirem convênios para a graduação e pós-graduação em enfermagem estabelecidos entre elas no que se refere aos campos de prática para o cuidado materno e infantil (Doc. 1; Doc. 2; Doc. 3; Doc. 7).

Servindo a uma população de aproximadamente 400 mil pessoas, a maioria de baixo poder aquisitivo, o HSF, localizado no Distrito Norte, na periferia de Belo Horizonte, capital de Minas Gerais, destaca-se entre várias maternidades existentes no Brasil por ter como propósito a humanização dos serviços para a mulher e a criança, com total colaboração da comunidade (Doc. 3).

Desde a sua fundação, o HSF contou com agentes históricos capazes de pensar sobre o cuidado ao indivíduo de forma audaciosa. Os diretores administrativos das décadas de 1980 e 1990 do HSF desenvolveram um trabalho que era uma grande novidade na política de saúde brasileira, tendo como sustentáculo do modelo de saúde a concepação comunitária baseada no tripé controle social exercido pela própria Associação Comunitária de Amigos e Usuários (ACAU) que levava humanização ao atendimento, pelo trabalho voluntário por meio de projetos como "mãe substituta" e "doula comunitária", mostrando o quanto a comunidade estava interessada em participar, e pela fiscalização, por meio da figura do "ombudsman" - a própria sociedade fiscalizando o seu hospital (Doc.1; Doc. 3).

Este modelo de saúde foi vanguardista e possibilitou que o HSF se tornasse cenário de grandes investimentos em diversos aspectos, a começar pela excelência de serviços na atenação à gestante e ao parto humanizado, o que acarretou uma melhora da qualidade da assistência prestada, e permitiu o alcance de títulos e prêmios, tais como Prêmio Galba de Araújo, concedido pelo Ministério da Saúde (MS), pela humanização à assistência ao parto (1990), "Primeiro Hospital Amigo da Criança" do estado de Minas Gerais, reconhecido pelo Fundo das Nações Unidas para a Infância (1995), e prêmio "Cidadania", conferido pelo Ministério da Saúde (1997) (Doc. 3). Foi também eleita a segunda melhor maternidade do Brasil (1998), sendo avaliado em relação a índice de cesarianas, humanização, atenação no pré-natal, infecação hospitalar, corpo clínico próprio, e estrutura de emergência e serviços de apoio (Doc. 2).

Estas conquistas estão ligadas a uma condiação visionária institucional no que diz respeito à valorização do trabalho multiprofissional ao ter uma assistência composta por enfermeira, médico, assistente social, psicólogo, farmacêutico, nutricionista e demais profissionais de enfermagem. E também por ter uma equipe plantonista formada por enfermeira e médicos obstetras, pediatra, anestesista e doula comunitária (Doc. 3).

A maternidade priorizou o enfoque de risco e o uso apropriado de tecnologias ao ofertar a assistência ao nascimento sob o ponto de vista holístico, interdisciplinar, e por considerar que não existe a concepação de doença e sim de vida, questionando o modelo tecnicista e biomédico presente em diversos hospitais mineiros (Doc. 3).

Com destaque para o conhecimento e as responsabilidades atribuídas à s enfermeiras do HSF, o hospital teve como princípio oferecer uma assistência mais humanizada na qual se resgata o caráter natural e fisiológico do nascimento, permitindo à mulher participar de forma ativa do processo. Uma das estratégias usadas para tal era deixar sob responsabilidade da enfermeira a assistência ao pré-natal e ao parto de risco (Doc. 3).

O modelo gestado e aperfeiçoado ao longo das décadas refletiu positivamente nos indicadores assistenciais. Nesse sentido, o HSF apresentou, desde 1982, a assistência ao parto realizada pela enfermeira em torno de 80% dos partos, e sistema de alojamento conjunto em 100% das internações. Desde 1988, foi centro de referência em planejamento familiar para o MS, e desde 1989 é cenário do Projeto Canguru no Brasil. Durante todas as internações, a mulher tem apoio do companheiro e de doula voluntária no pré-parto, parto e pós-parto (Doc. 3; Doc. 4). A maternidade tinha uma média de 400 partos/mês, sendo 15% eram pacientes de risco (Doc. 3), e enfrentava desafios em relação à taxa crescente de cesarianas, o qual se aproximava a extrapolar o percentual preconizado pelo MS (Doc. 6).

A partir do momento em que o HSF foi se tornando referência na assistência à gestante, surge também a necessidade de continuidade do cuidado ao recém-nascido de risco. Até aquele momento, o HSF contava com leitos de pediatria, e desde 1998 estava em andamento um projeto para a criação de um berçário de alto risco, nos moldes da internação conjunta (Doc. 1; Doc. 3). Até o ano de 1999, os recém-nascidos que precisavam de assistência crítica eram referenciados para o Hospital Odilon Behrens. Diante dos indicadores, isto tinha alto custo e poderia trazer maiores prejuízos à s mães e crianças referenciadas, indo contra o modelo de saúde proposto e humanização defendido pela instituiação (Doc. 1; Doc. 6).

Diante das proporções de desenvolvimento do HSF, surgiu a necessidade de o hospital ter sob sua responsabilidade os cuidados de todos os pacientes neonatais, uma vez que o convênio com o Hospital Odilon Behrens possuía um custo mais elevado e gerava mais custos para referenciar todos os pacientes (Doc. 6).

Diante da qualidade de saúde que estava sendo prestada pelo HSF à população mineira, a prefeitura de Belo Horizonte, por meio do convênio com o HSF, entregou à comunidade 10 leitos de Unidade de Cuidados Intermediários Neonatal no HSF, leitos estes destinados aos recém-nascidos que precisavam de um cuidado especializado, configurando-se o primeiro espaço destinado ao cuidado especializado ao recém-nascido de risco (Doc. 5).

Naquele cenário, a EEUFMG, uma instituiação com quase 70 anos, manteve-se protagonista dos principais processos de profissionalização da enfermagem mineira, em busca da construação e produação de um conhecimento próprio para a profissão e, mesmo sendo influenciada pelo contexto político, econômico e pela necessidade de legislação específica, a EEUFMG procurou atender à s demandas de Minas Gerais em relação à saúde neonatal (Doc. 7).

### Dando vida as ideias : implantação do curso de Especialização em Enfermagem hospitalar

A EEUFMG propôs a criação do Curso de Especialização em Enfermagem Hospitalar (CEEH), abrangendo como princípio o aprendizado em serviço, e tendo como intenação contribuir com a formação de recursos humanos de enfermagem para a área hospitalar e, ao mesmo tempo, refletir sobre a sua prática assistencial aos pacientes neonatais (Doc. 7).

O CEEH teve sua metodologia baseada nos pressupostos da pedagogia da problematização. O curso foi oferecido na modalidade semipresencial, com momentos de concentração e momentos de dispersão, de modo a considerar a experiência e o cotidiano do trabalho do enfermeiro orientada nos pressupostos da articulação ensino/trabalho (Doc. 7).

O curso foi orientado por diferentes técnicas de ensino, tais como seminários, conferências, aulas expositivas, trabalhos em pequenos grupos, discussões de casos clínicos e gerenciais, e estudos independentes, buscando a participação ativa do aluno. As atividades de estudos independentes compreenderam leitura, análise de bibliografia e atividades e práticas no campo de trabalho de cada aluno, em campo previamente definido (Doc. 7).

O corpo docente era composto por vinte e três enfermeiras, sendo que sete possuíam título de doutora, quinze detinham o título de mestra e uma tinha o título de especialista. Todas as docentes tinham atividades voltadas para o ensino do curso - dezoito tinham atividades relacionadas somente à orientação, e dezoito eram vinculadas ao ensino e à orientação dos discentes (Doc. 7).

Os docentes que constituíram o curso eram, em sua maioria, egressos do curso de mestrado em enfermagem da UFMG (Doc. 7), o que traz uma maior vinculação com a instituiação e compromisso com o avanço do conhecimento próprio da enfermagem mineira.

O CEEH possuía carga horária de 420 horas, o que correspondia a 28 créditos, e deveria ser desenvolvido em no máximo doze meses. Sua administração era feita pelo Colegiado de Pós-Graduação da EEUFMG, e tinha seu corpo de docentes pertencente aos três departamentos da EEUFMG, a saber: Enfermagem Básica; Enfermagem Aplicada, e Enfermagem Materno Infantil e Saúde Pública, o que demonstra uma confluência dos diversos saberes que estes docentes compunham.

A enfermeira que optasse pela área de concentração em neonatologia teria sua grade curricular organizada em dois momentos, sendo um tronco comum e um tronco específico. O tronco comum tinha uma carga horária teórica de 210 horas e compreendia um conjunto de conteúdos necessários ao perfil do enfermeiro especialista no campo pedagógico, clínico e gerencial. Era composto por cinco módulos, sendo eles: Capacitação pedagógica, Avaliando a situação de saúde do cliente, Metodologia da Pesquisa, Rompendo a cadeia de transmissão de doenças, e Gerenciando o Cuidado de Enfermagem (Doc. 7).

Estas disciplinas juntas abarcavam um conjunto de conhecimentos capazes de promover o desenvolvimento das atividades de ensino-aprendizagem do curso, e contribuíam para a sistematização da aplicação do conhecimento na atividade profissional do enfermeiro e futuro especialista na área de neonatologia (Doc. 7; Doc. 8).

O tronco específico era "Enfermagem em Neonatologia" e tinha o HSF como cenário da prática assistencial. Compreendia um conjunto de conhecimentos teóricos em neonatologia com aprofundamento de metodologias de assistência que respaldavam o aluno na área de escolha. O tronco específico contava com uma carga horária total de 210 horas (Doc. 7; Doc. 8).

Em sua ementa, o curso com à ªnfase em Enfermagem em Neonatologia buscou abordar as características e as necessidades biopsicossociais do recém-nascido e de suas famílias, bem como as demandas para o cuidado integral de enfermagem (Doc. 7; Doc. 8). Tinha como objetivo analisar os agravos mais frequentes que acometiam os recém-nascidos, destacando a fisiopatologia e as medidas de intervenação para aplicar os cuidados de enfermagem ao recém-nascido a termo, pré e pós-termo, na sala de parto, no alojamento conjunto, na unidade de neonatologia, bem como o seu acompanhamento ambulatorial (Doc. 7; Doc. 8).

Nesta perspectiva, quando os três departamentos de enfermagem da EEUFMG se uniram para a oferta de um curso de especialização, eles estavam buscando e investindo em uma formação interdisciplinar do enfermeiro. Este investimento resultou na formação de dez enfermeiras especialistas em neonatologia e no pioneirismo da EEUFMG na oferta de pós-graduação em enfermagem neonatológica em Minas Gerais.

## DISCUSSÃO

Este estudo utilizou o referencial da Sociologia das Profissões, de Eliot Freidson^(15,16)^. A construação histórica de um campo de conhecimento para a enfermagem neonatológica perpassa diversas lutas firmadas pela categoria. Dessa maneira, é importante citar que as profissões são ocupações que assumem posições dominantes na divisão do trabalho e controlam e determinam a essência do próprio trabalho, sendo, portanto, autônomas e autorreguladas^(15,16,21)^.

O autor estabelece que a expansão e consolidação de uma profissão acontece por meio da sua organização profissional e do profissionalismo dos membros constituintes dessa atividade laboral formal. Dessa forma, a profissionalização de uma disciplina caracteriza-se por três fatores: autonomia, *expertise* e credencialismo^(15,16,22)^.

A autonomia compreende-se como o domínio de uma profissão no controle do aspecto técnico do seu trabalho, estabelecendo aquilo que é a essência própria e única da profissão. Este fator torna-se evidente em ambientes em que há divisão do trabalho entre as profissões, e cada área possui suas especificidades e atribuições próprias^(15,16,21,22)^.

A autonomia da enfermeira do Hospital Sofia Feldman (HSF) é uma aposta institucional. Desde a sua gênese, a instituiação esteve atenta à s necessidades de saúde da população, e confiava o cuidado do pré-natal e ao parto de risco a "enfermeiras". Desta forma, é importante considerar que o alcance da autonomia depende de certos fatores, como a habilidade de tomar decisões independentes, ser livre de coeração, ter pensamento racional e reflexivo, conhecimento e informações adequadas, além do direito de controlar o próprio trabalho^(23,24)^.

Os escritos de Freidson apontam a Enfermagem como uma ocupação ou mesmo como atividade paraprofissional^(15,16)^, que depende das determinações de outro profissional para exercer a sua prática^(24)^. Na visão de Freidson, tradicionalmente, a maioria dos enfermeiros não consegue ter autonomia pelo fato de trabalharem em um hospital pautado no modelo biomédico, já que muitos dependem das ordens e exigências médicas, aumentando, assim, o potencial de conflitos^(15,16)^. Não se concorda, contudo, com esta assertiva, entendendo que a tomada de decisão para a criação do Curso de Especialização foi um exercício que implicou em autonomia, credencialismo e *expertise* das organizadoras em conseguir esta autorização e implantarem o curso com galhardia.

Quando se analisa o cenário da década de 1990, o HSF foi uma instituiação vanguardista que acreditou e apostou no trabalho da enfermeira. Tornou-se, desta forma, um lugar que viabilizava e fomentava espaços de resistências capazes de enfraquecer a hegemonia tradicional e possibilitar que as "enfermeiras" que ali trabalhavam tivessem a sua governabilidade reinventada e autonomia fomentada, o que garantia uma assistência segura e qualificada.

Alguns estudos apontam que a autonomia profissional da enfermeira no ambiente de terapia intensiva neonatal ainda é limitada e, por vezes, anulada no interior das organizações de saúde, como por exemplo em instituições onde o modelo biomédico é fortemente exercido, nesses locais, a autonomia profissional da enfermeira é cerceada e condicionada pelas decisões do profissional médico, pela frágil construação de um corpo de saberes próprio à profissão, e pela crescente divisão técnica do trabalho em saúde e em enfermagem^(25-28)^.

No HSF, a autonomia da enfermeira era incentivada nos espaços coletivos da instituiação, a começar pela existência de uma equipe multiprofissional e interdisciplinar composta por enfermeira, médico, assistente social, psicólogo, farmacêutico, nutricionista e demais profissionais de enfermagem, e também de uma equipe plantonista formada por enfermeira e médicos obstetras, pediatra, anestesista e doula comunitária. Essa composiação de equipe permitiu e contribuiu para que a enfermeira, a partir do credencialismo assegurado pelo Estado e pelos órgãos profissionais, se tornasse instrumento de autonomização do Hospital Sofia Feldman. Cabe ressaltar que a realização destas atividades pelo HSF se deve ao entendimento de que as estratégias capazes de modificar e de qualificar as práticas em saúde são construídas a partir do diálogo entre cada um dos atores envolvidos na atenação ao parto e nascimento, e na convivência colaborativa nas diferenças das profissões.

A expertise é o conhecimento próprio da profissão que fornece a essência formal do aprendizado dos *experts* em escolas profissionais, sendo conformada por formação específica submetida a regulamentos formais^(15,16,21,22)^. Neste sentido, quando foi criado o CEEH com à ªnfase na enfermagem neonatológica, o objetivo era forjar um saber específico para atuação em lócus determinado.

O HSF é um desses espaços, um hospital público específico e referência no cuidado à mulher e ao recém-nascido, nos seus diversos modos de cuidado^(29)^. A escolha deste cenário como campo de prática de um curso de especialização que formava enfermeiras neonatais tinha como objetivo dar conhecimento ao enfermeiro para atuar diretamente nas reais necessidades de saúde da população, e melhorar as condições profissionais de atuação, ou seja, formar um grupo de *experts* em neonatologia que servisse de molas propulsoras para a formação de outros profissionais. O CEEH tinha como preocupação preparar o enfermeiro para o contato com a população. Esta preocupação é reforçada pelo fato de a instituiação de escolha deter o título internacional "Hospital Amigo da Criança" desde 1995, um modelo de saúde baseado na concepação comunitária, e um de seus três pilares sendo pautado no controle social^(29)^. As disciplinas "Capacitação pedagógica" e "Avaliando a situação de saúde do cliente" mostravam essa abordagem em 75 horas de aula teórica.

Outrossim, a expertise se expressa no profissional de consulta, que detém, de algum modo, um monopólio sobre o exercício dos seus saberes próprios, e tem a tarefa de resolver problemas práticos apresentados pela sociedade, atraindo sua confiança espontânea em consultá-lo^(15,16,22)^. Não se pode deixar de mencionar que esse conhecimento permitiu o reconhecimento do *status quo* dessa enfermeira junto à população assistida, fazendo com que o profissional seja autônomo e legitimado pelas credenciais conferidas.

Assim, a *expertise* garante a especificidade do saber-fazer, e confere autonomia no controle da definiação de problemas sob o domínio do profissional. Além disso, cria condições para a estruturação e organização de bases para o exercício autônomo da profissão^(21,23,24)^.

Sendo assim, a *expertise* produzida nos ambientes de produação de conhecimento confere aos pacientes neonatais uma proteação relacionada ao cuidado especializado prestado pela enfermeira, conforme demonstra a literatura^(30,31)^. A construação de conhecimento científico em neonatologia como campo de conhecimento da enfermeira auxiliou na conquista de espaços na instituiação e na profissão, que foram fatores importantes para o alcance de títulos e honrarias devidos à qualidade de saúde que estava sendo produzida pelas "enfermeiras" do HSF.

Ressalta-se o papel da EEUFMG, protagonista de processos de profissionalização no estado e no Brasil, ao ser pioneira por conduzir mais uma frente de trabalho em prol da autonomização da enfermeira^(5)^, tendo como sustentáculo o saber próprio para o atendimento das demandas sóciossanitárias. Destaca-se o HSF pelas ações inovadoras no setor da saúde, e o envolvimento com a comunidade que possibilitou o desenvolvimento de ações voltadas para as necessidades reais da população.

### Limitações do estudo

Trata-se de um estudo circunscrito a um cenário de uma capital brasileira, que tem como instituições um hospital maternidade e uma escola de enfermagem que são referências para o estado. A interpretação de fontes de outras localidades poderia denotar circunstâncias de saúde e de formação de profissionais de saúde que não são necessariamente aquelas vividas em grandes centros. Ademais, a não realização da busca em outros acervos, como por exemplo da Biblioteca Pública, pode não ter permitido contemplar todas as informações acerca da formação das enfermeiras em neonatalogia no período estudado.

### Contribuições para a área da saúde, da enfermagem ou de políticas públicas

Trata-se do primeiro investimento de pesquisa historiográfica da enfermagem neonatal no estado de Minas Gerais. Os resultados encontrados elucidam fatos históricos que apoiaram o entendimento da trajetória de protagonismos da EEUFMG e do HSF no contexto da profissionalização da enfermagem mineira e brasileira, contribuindo para registro e visibilização de um modelo pioneiro de formação de enfermeiras engajado com as necessidades de saúde da população.

A partir da publicização da configuração de um campo de conhecimento especializado em enfermagem no estado de MG, contribui-se para o reconhecimento das enfermeiras, sujeitos históricos delineadores do processo de profissionalização, gerando o pertencimento e uma identidade profissional coletiva que se une em um corpus de conhecimento e na luta pela apropriação e defesa deste espaço.

A historiografia da criação e implantação do serviço de enfermagem em um cenário especializado, para além da preservação da memória, também fortalecerá a identidade profissional e, por conseguinte, institucional, pois a experiência histórica de um grupo ou sociedade fornece referências para o presente. A historiografia da saúde apresenta um conjunto de pesquisas^(29-31)^, porém é importante destacar o ineditismo desta pesquisa no cenário da enfermagem neonatológica em Minas Gerais e no contexto nacional.

## CONSIDERAÇÕES FINAIS

A primeira oferta de um curso para a formação de enfermeiras especialistas em neonatologia pela EEUFMG, em parceria com o HSF, representou um marco na história da enfermagem mineira, e de ambas as instituições. O curso contribuiu para o avanço da profissão, implantação de práticas inovadoras, e autonomização da profissional enfermeira na construação do conhecimento próprio de enfermagem, possibilitando a obtenação do conhecimento especializado e das credenciais necessárias para amparar a reserva de mercado de trabalho.

## Data Availability

Os dados de pesquisa só estão disponíveis mediante solicitação.
